# Activation of adiponectin receptors has negative impact on muscle mass in C2C12 myotubes and fast-type mouse skeletal muscle

**DOI:** 10.1371/journal.pone.0205645

**Published:** 2018-10-11

**Authors:** Rika Ito, Masaki Higa, Ayumi Goto, Megumi Aoshima, Akihiro Ikuta, Kazuya Ohashi, Shingo Yokoyama, Yoshitaka Ohno, Tatsuro Egawa, Hirofumi Miyata, Katsumasa Goto

**Affiliations:** 1 Biological Sciences, Graduate School of Sciences and Technology for Innovation, Yamaguchi University, Yamaguchi City, Yamaguchi, Japan; 2 Department of Physiology, Graduate School of Health Sciences, Toyohashi SOZO University, Toyohashi, Aichi, Japan; 3 Sportology Center, Graduate School of Medicine, Juntendo University, Tokyo, Japan; 4 Laboratory of Physiology, School of Health Sciences Toyohashi SOZO University, Toyohashi, Aichi, Japan; 5 Laboratory of Sports and Exercise Medicine, Graduate School of Human and Environmental Studies, Kyoto University, Kyoto City, Kyoto, Japan; University of Minnesota Medical Center, UNITED STATES

## Abstract

This study investigated the effects of AdipoRon, which is an agonist for adiponectin receptor 1 (AdipoR1) and AdipoR2, on the protein content, myotube diameter, and number of nuclei per myotube of C2C12 cells and skeletal muscle mass in C57BL/6J mice. AdipoRon suppressed the protein content, myotube diameter, and number of nuclei per myotube of C2C12 cells of C2C12 myotubes in a dose-dependent manner. Adiponectin-associated decline of protein content, diameter, and number of nuclei per myotube in C2C12 myotubes was partially rescued by knockdown of AdipoR1 and/or AdipoR2. Phosphorylation level of AMPK showed a trend to be increased by AdipoRon. A significant increase in phosphorylation level of AMPK was observed at 20 μM AdipoRon. Knockdown of AdipoR1 and/or AdipoR2 rescued AdipoRon-associated decrease in protein content of C2C12 myotubes. AdipoRon-associated increase in phosphorylation level of AMPK in C2C12 myotubes was suppressed by knockdown of AdipoR1 and/or AdipoR2. Successive intravenous injections of AdipoRon into mice caused a decrease in the wet weight of plantaris muscle (PLA), but not in soleus muscle (SOL). Mean fiber cross-sectional area of PLA, but not of SOL, was significantly decreased by AdipoRon administration. On the one hand, the expression level of phosphorylated AMPK and ubiquitinated protein in SOL and PLA muscles was upregulated by AdipoRon administration. On the other hand, AdipoRon administration induced no changes in the expression level of puromycin-labeled proteins in both SOL and PLA muscles. Expression level of adiponectin in extensor digitorum longus (EDL) muscle was increased by aging, but not in SOL muscle. Aging had no effect on the expression level of AdipoR1 and AdipoR2 in both muscles. Phosphorylation level of AMPK in EDL was increased by aging, but not SOL muscle. Results from this study suggest that high level of circulating adiponectin may induce skeletal muscle atrophy, especially fast-type muscle.

## Introduction

Skeletal muscle has a large plasticity. Skeletal muscle mass as well as function adapts to various extracellular and intracellular stimuli. Skeletal muscle atrophy is induced by inactivity, unloading, malnutrition as well as several diseases, including cancer, diabetes and heart failure [1,2]. Sarcopenia is well known as an aging-associated decline in skeletal muscle weakness accompanied with mass loss and dysfunction, resulting in impaired physical function [3]. Reduced physical function leads to physical inactivity that induces obesity, osteoporosis, frailty, and other various life style-related diseases [4]. Therefore, the maintaining of skeletal muscle mass and function is a priority task in most countries with increasing populations have increasing percentages of large shares of adults aged 65 years and older, due to rising health-care cost [5,6]. However, the molecular mechanisms for aging-associated reductions in skeletal muscle mass remains unclear.

Basic experiments revealed that adiponectin is an adipocytokine that have various anti-pathophysiological action such as insulin sensitization, anti-inflammatory, stimulating effects of energy utilization, and so on. Adiponectin, which is synthesized and is secreted by adipocytes, induces physiological functions in targeted tissues and cells via adiponectin receptors (AdipoRs) [7,8]. Skeletal muscle, which is a main target tissue for adiponectin, expresses high level of AdipoR1 and low level of AdipoR2, which is main expressing form of hepatocytes [9].

Recently, there are several epidemiological studies regarding negative relationship between circulating adiponectin level and mortality rate as well as various clinical conditions, so-called “adiponectin paradox” [10,11]. For instance, circulating level of adiponectin is negatively correlated with skeletal muscle mass as well as force [12,13,14]. However, there are no experimental evidence showing the effects of elevated blood adiponectin level on skeletal muscle mass.

Not only AdipoRs but also adiponectin itself expresses in skeletal muscle cells [15,16]. In addition, it is suggested that skeletal muscle cells themselves synthesize and may secrete adiponectin in an autocrine- and/or paracrine-manner [15]. If high level of adiponectin may impact on skeletal muscle mass, the expression level of adiponectin and/or AdipoRs in skeletal muscle also could be a key factor to regulate skeletal muscle mass. There is no evidence regarding aging-associated changes in the expression level of adiponectin and AdipoRs in skeletal muscles.

Skeletal muscle mass is regulated by a dynamic balance between protein synthesis and proteolysis. Hypertrophic stimuli such as mechanical stretch, cause to increase in protein synthesis, resulting the increase in muscle protein and mass, which is namely muscle hypertrophy [17,18]. On the other hand, unloading stimulates proteolysis in skeletal muscle cells, and induces the decline of muscle protein and mass, namely muscle atrophy [1,15,19]. Although there are many evidences regarding the dose-dependent effects of adiponectin on insulin sensitivity and AMP-dependent protein kinase (AMPK) that is activated by adiponectin [20,21], the effect of various concentration of adiponectin on protein synthesis and proteolysis in skeletal muscles remain unclear.

Therefore, in this study, we investigated the administration of AdipoR agonist AdipoRon on muscle mass using cultured skeletal muscle cells and adult mouse skeletal muscles to clarify whether a high level of adiponectin induces skeletal muscle atrophy or not. Effects of aging on the expression level of adiponectin and AdipoRs in mouse skeletal muscles were also investigated.

## Materials and methods

Mouse myoblast-derived cell-line C2C12 was used in the cell culture experiment. All animal protocols were carried out in accordance with the Guide for the Care and Use of Laboratory Animals as adopted and promulgated by the National Institutes of Health (Bethesda, MD) and were approved by the Animal Use Committee at Toyohashi SOZO University (A2014002, A2015002, A2016004, A2017007). All treatments of animals were performed under anesthesia with *i*.*p*. injection of sodium pentobarbital, and all efforts were made to prevent discomfort and suffering. Young (10-week old) and old (100-week-old) male C57BL/6J mice were used. All mice were housed in a vivarium room with 12:12-h light:dark cycle and a maintained temperature and humidity of ~23°C and ~50%, respectively. Solid food and water were provided *ad libitum*.

### Cell culture experiments

C2C12 myoblasts were cultured on culture plates coated with a genetic type I collagen (BioCoat, 12 wells, Corning, NY). Cells were grown in the growth medium consisting of Dulbecco's modified Eagle's medium (DMEM) supplemented with 10% heat-inactivated fetal bovine serum containing high glucose (4,500 mg glucose/L) in a humidified atmosphere of 95% air and at 37°C in 5% CO2 for 24 h (~80% confluent).

#### RNA interference

Twenty-four hours after the seeding, RNA oligos were transfected into myoblasts using Lipofectamine^TM^ RNAiMAX Transfection Reagent (Thermo Fisher Scientific, Waltham, MA) according the manufacturer’s instructions. Details for the transfection of sRNA was described previously [22]. Briefly, lipofectamine/siRNA complexes were added the growth medium, and myoblasts were incubated for 24 h. The final concentration of siRNA was set at 5 nM in a single knockdown of AdipoR1 or AdipoR2 and 10 nM in a double knockdown of AdipoR1 and AdipoR2, respectively. Following 24-h incubation, the transfection medium was changed with the differentiation medium consisting of DMEM supplemented with 2% horse serum containing low glucose (1,000 mg/L glucose). The culture medium was then changed to a differentiation medium. Differentiation medium was exchanged every other day.

The siRNA oligonucleotides designed against mouse adiponectin receptor 1 (siAdipoR1: targeting sequence 5’-CAGGGATTGCTCTACTGATTA-3’), siAdipoR2 (targeting sequence 5’-CAGGCCCATCATGCTATGGAA-3’), and scrambled non-targeting control siRNA (siScramble: All Stars Negative Control siRNA) were obtained from Qiagen (Hiden, Germany). Knockdown efficiency of each siRNA was checked using Real-time RT-PCR. mRNA Expression level of for AdipoR1 and/or AdipoR2 was decreased by approximately 76~90% (S1 Fig). Positive control experiment was also carried out using siRNA for glyceraldehyde 3-phosphate dehydrogenase (GAPDH) supplied by Thermo Fisher Scientific (4390849: Silencer^TM^ Select GAPDH Positive Control siRNA). GAPDH mRNA was decreased by ~90% in the knockdown procedure in this experiment using siRNA (S2 Fig).

#### AdipoRon treatment

We investigated the effects of AdipoRon (AdipoGen Life Sciences, San Diego, CA), instead of adiponectin, on C2C12 myotubes. AdipoRon binds AdipoRs, and induces adiponectin-associated physiological functions via AMPK-associated intracellular signaling [20,23]. Five days after the initiation of differentiation, AdipoRon was added to the differentiation medium. C2C12 myotubes were incubated with AdipoRon for 36 h. AdipoRon was dissolved in dimethyl sulfoxide (DMSO). Final concentrations of AdipoRon in the medium was set at 0, 5, 10, and 20 μM. After 36 h-incubation of AdipoRon, cells were collected.

### Animal experiments

#### Effects of AdipoRon administration on mouse skeletal muscles

We investigated the effects of AdipoRon administration on skeletal muscle mass in C57BL/6J male mice (10-week old). Mice were divided into 2 groups; control (n = 7) and AdipoRon-administrated (n = 5) groups.

AdipoRon (50 mg/kg body weight) was dissolved in DMSO (0.75 μl/g body weight), and was injected into caudal vein of mice in the AdipoRon-administrated group, in accordance with the previous study [23]. Although we did not evaluate the serum concentration of AdipoRon, this amount of AdipoRon could induce various physiological actions similar to adiponectin [23]. AdipoRon was injected once a day, 3 days a week, for 4 weeks. DMSO (0.75 μl/g body weight) was injected into mice of the control group. Three days after the final injection, soleus (SOL) and plantaris (PLA) muscles were dissected from both hindlimbs of mice in both groups. Muscles were trimmed of excess fat and connective tissues, weighed, frozen in liquid nitrogen, and stored at −80°C.

#### Expression level of adiponectin and its receptor in skeletal muscles in young and old mice

We also investigated the effects of age on skeletal muscle mass in young (10-week old, n = 5) and old (100-week old, n = 5) C57BL/6J male mice. SOL and extensor digitorum longus (EDL) muscles were dissected from both hindlimbs of young and old mice. Muscles were trimmed of excess fat and connective tissues, weighed, frozen in liquid nitrogen, and stored at −80°C.

### Sample preparation

In the cell culture experiments, sample preparation was performed with the previously reported method [15,22]. Briefly, the cells in each well were scraped off into 0.15 ml of the lysis buffer that consists of CelLytic^TM^-M cell lysis reagent (Sigma, St. Louis, MO) with 1% (v/v) protease/protease inhibitor cocktail (Cell Signaling Technology, Danvers, MA). The cell lysate was sonicated and centrifuged at 15,000 g at 4°C for 15 min. Then, the supernatant was collected.

In the animal experiments, the frozen muscle samples were homogenized in 5 volumes of the lysis buffer with glass homogenizer as described as previously [15]. The homogenate was centrifuged at 15,000 g at 4°C for 15 min. The supernatant was collected.

Protein content of the supernatant from both cells and skeletal muscles was evaluated using the Bradford technique (protein assay kit; Bio-Rad with bovine serum albumin (Sigma) as the standard [15,22].

### Evaluation of protein synthesis

Surface sensing translation (SUnSET) is a nonradioactive method to monitor and quantification of global protein synthesis in mammalian cells [24]. Briefly, 30 min before sampling, mice were injected intraperitoneally with puromycin (0.04 μmol/g) dissolved in 100 μl of phosphate-buffered saline under anesthesia. The expression of puromycin-labeled proteins was analyzed by Western blot analyses as described below.

### Evaluation of proteolysis

In the present study, the proteolysis in skeletal muscle was evaluated the expression level of ubiquitinated proteins using Western blot analyses as described below.

### Western blot analysis

Western blot analysis was performed, as was described previously [15,22]. Extracted samples in the cell lysis reagents were solubilized in Laemmli's sample buffer and boiled for 5 min. The samples were separated by SDS-PAGE using 10 or 15% polyacrylamide gel at a constant current of 20 mA/gel for 90 min. Bio-Rad Precision Markers (Bio-Rad Laboratories, Hercules, CA) were applied to both sides of gel as the internal controls for transfer process or electrophoresis.

After SDS-PAGE, the proteins were transferred to polyvinylidene fluoride membranes (Hybond-P, GE Healthcare, Buckinghamshire, UK) by using trans-blot cell (Bio-Rad) at a constant voltage of 100 V for 1 h at 4°C. After the transfer, the membranes were blocked for 1 h using ECL blocking reagent (RPN418, GE Healthcare). For the analyses on the expression level of ubiquitin- and puromycin-labeled proteins, the membranes were stained with Ponceau S solution (Sigma). Then, the membranes were incubated over night at 4°C with primary antibody [adiponectin (ab22554, Abcam, Cambridge, UK), AdipoR1 (GTX104770, GeneTex, Irvine, CA), AdipoR2 (14361-1-AP, Proteintech, Rosemont, IL), AMPKα Thr^172^ (2531, Cell Signaling Technology), AMPK (2532, Cell Signaling Technology), ubiquitin (SPA-200, Enzo Life Sciences, NY), puromycin (MABE343, Merck, Darmstadt, Germany), glyceraldehyde 3-phosphate dehydrogenase (GAPDH: 2118, Cell Signaling Technology) and β-actin (4967, Cell Signaling Technology)]. The membranes were then washed with Tris-buffered saline with 0.1% Tween 20 (TBS-T, pH 7.5) and reacted with anti-rabbit IgG (7074, Cell Signaling Technology), anti-mouse IgG (7076, Cell signaling Technology) or anti-mouse IgG2a (ab98698, abcam) for 1h at room temperature. After the final wash with TBS-T, protein bands were visualized using chemiluminescence (GE Healthcare), and signal density was measured using Light-Capture (AE-6971, ATTO Corporation, Tokyo, Japan) with ImageJ software (National Institutes of Health, MD).

### Real-time RT-PCR analysis

Real-time RT-PCR analysis was performed, as was described previously [15,21]. Briefly, total RNA was extracted from muscles using the miRNeasy Mini kit (Qiagen) according to the manufacturer’s protocol. For the detection of AdipoR1 and AdipoR2, the RNA was reverse-transcribed to cDNA using PrimeScript RT Master Mix (Takara Bio, Otsu, Japan), and then synthesized cDNA was applied to real-time RT-PCR (Thermal Cycler Dice Real Time System IIMRQ, Takara Bio) using Takara SYBR Premix Ex Taq II (Takara Bio). Relative fold change of expression was calculated by the comparative CT method with Takara Thermal Cycler Dice Real Time System Software Ver. 4.00. To normalize the amount of total RNA present in each reaction, GAPDH for AdipoR1 and AdipoR2 were used as an internal standard.

Following primers were used: adiponectin, 5’-TTCTGTCTGTACGATTGTCAGTGG-3’ (forward) and 5’-GTCATCTTCGGCATGACTGG-3’ (reverse), AdopoR1, 5’-CTGGGCATCTCTGCCATCA-3’ (forward) and 5’-CTTGACAAAGCCCTCAGCGATA-3’ (reverse); AdipoR2, 5’-ATCAGCAGCCAGACGACTC-3’ (forward) and 5’-TGACCAGTCCCAAAGACCTCTACTC-3’ (reverse); GAPDH, 5’-TGTGTCCGTCGTGTGGATCTGA-3’ (forward) and 5’-TTGCTGTTGAAGTCGCAGGAG-3’ (reverse).

### Morphological analyses of myotubes

Myotube diameter was evaluated as described earlier [22]. Briefly, 4 fields were chosen randomly, and 150 myotubes were measured using ImageJ. The average diameter per myotube was calculated as the mean of three short-axis measurements taken along the length of the myotube. To evaluate differentiation level of myotubes, the number of nuclei per myotube was also investigated.

### Histochemical and immunohistochemical analyses

Serial transverse cryosections (7-μm thick) of frozen distal portion of SOL and PLA muscles were cut at -20 ^o^C and mounted on the slide glasses. The sections were air-dried and stained to analyze the degree of muscle damage and repair and the cross-sectional area (CSA) of muscle fibers by staining using hematoxylin and eosin (H&E) [25,26].

### Statistical analyses

**Fig 1:**
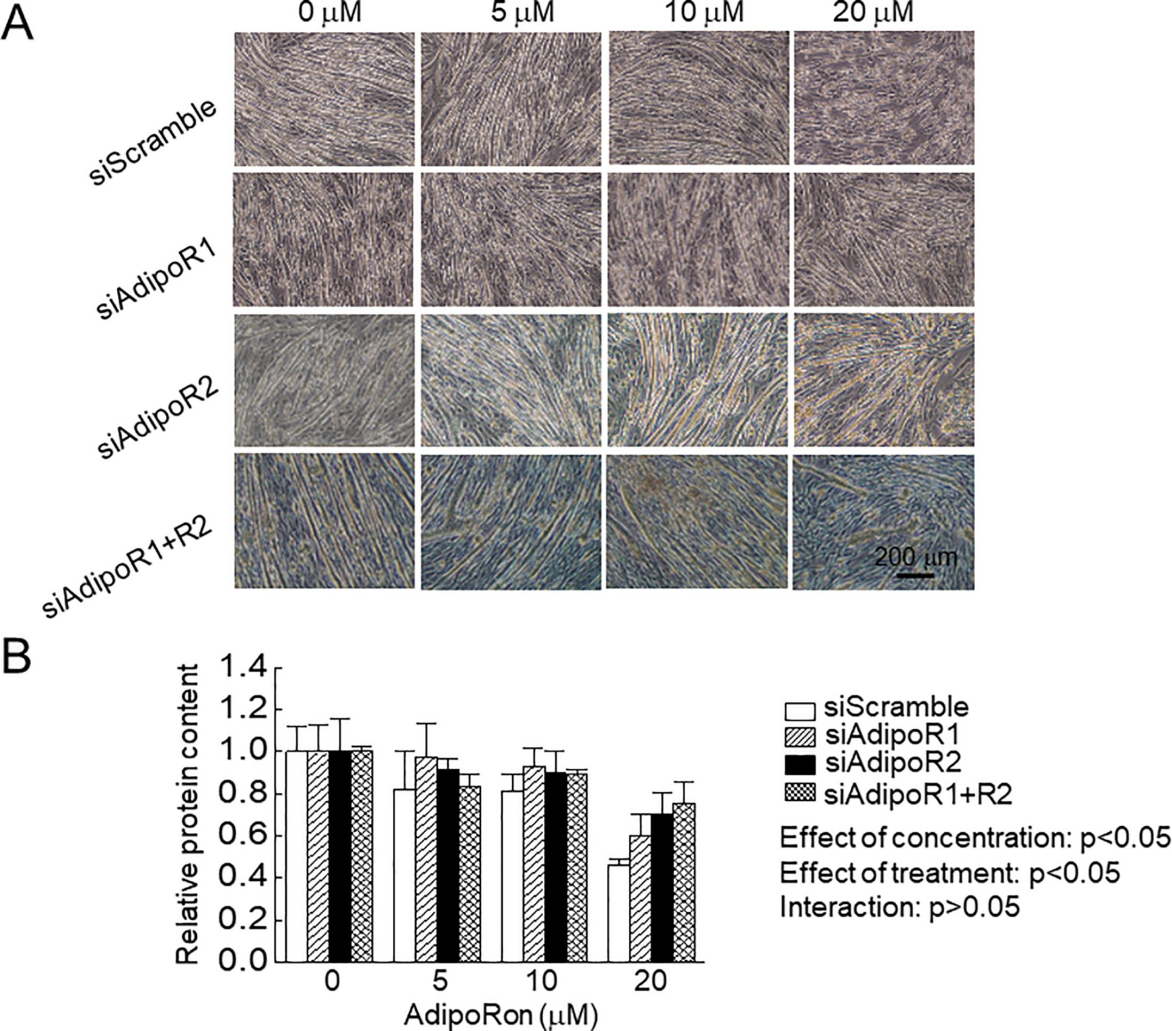
Effects of AdipoRon administration on C2C12 myotubes with or without knockdown of adiponectin receptor (AdipoR). Final concentration of AdipoRon was set at 0, 5, 10 and 20 μM in the differentiation medium. A: Effects of AdipoRon on C2C12 myotubes morphology with or without AdipoR knockdown. B: Effects of AdipoRon on protein content in C2C12 myotubes with or without AdipoR knockdown. siScramble: scrambled non-targeting control siRNA, siAdipoR1: siRNA for AdipoR1, siAdipoR: siRNA for AdipoR2, siAdipoR1+R2: siRNA for AdipoR1 and AdipoR2. n = 5 in each condition of each treated cells. Values are expressed means with SEM. *: p<0.05. Significant level of main effects was analyzed by two-way ANOVA (concentration x treatment).

**Fig 2:**
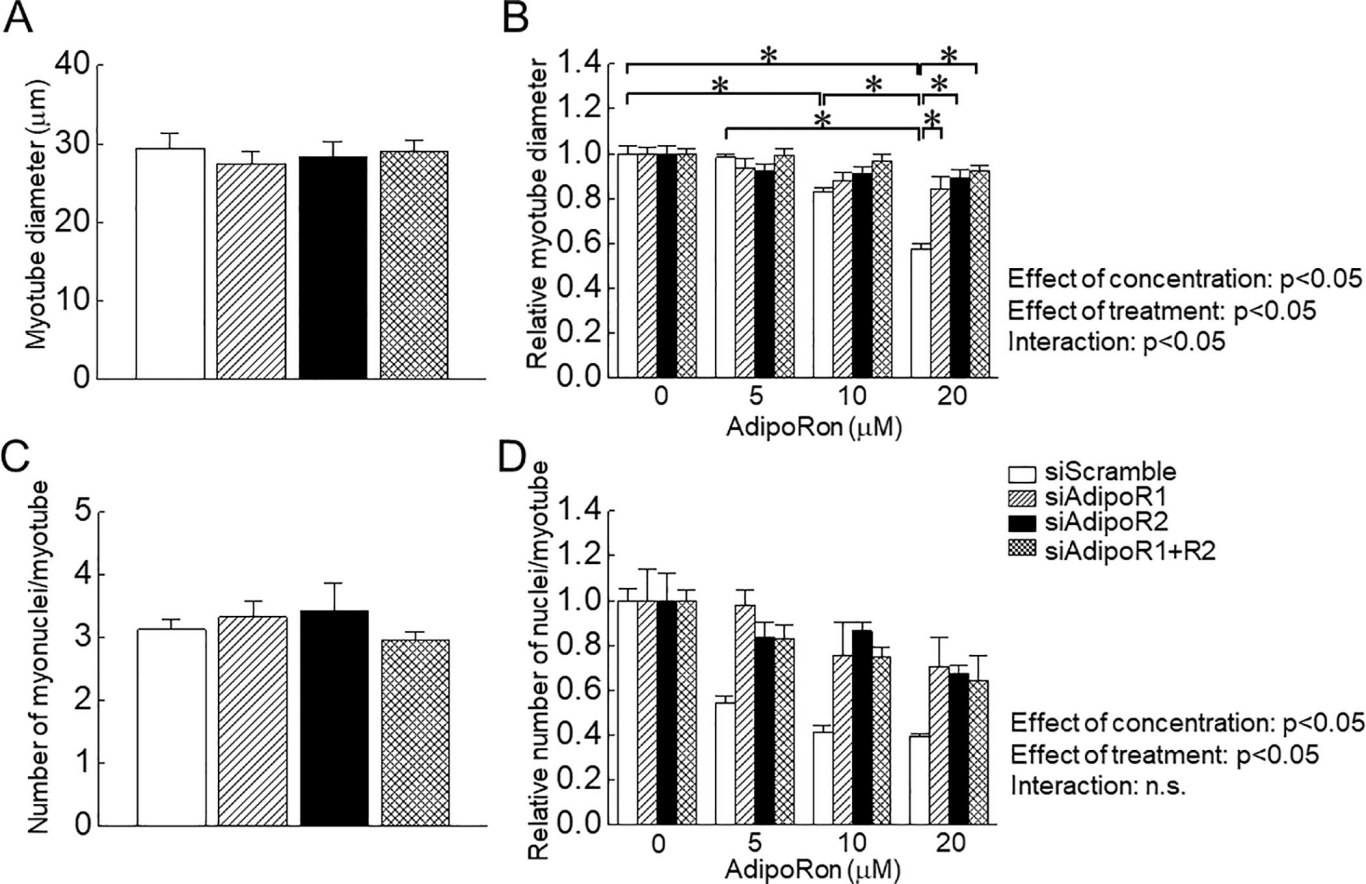
Effects of AdipoRon administration on myotube diameter and number of nuclei per myotube of C2C12 cells with or without knockdown of adiponectin receptor (AdipoR). Final concentration of AdipoRon was set at 0, 5, 10 and 20 μM in the differentiation medium. A: Effects of AdipoR knockdown on myotube diameter at 0 μM AdipoRon. B: Effects of AdipoRon on the relative myotube diameter with or without AdipoR knockdown. C: Effects of AdipoR knockdown on the number of nuclei per myotube at 0 μM AdipoRon. D: Effects of AdipoRon on the relative number of nuclei per myotube with or without AdipoR knockdown. Abbreviations are the same as in Fig 1. Values are expressed means with SEM. *: p<0.05. Significant level of main effects was analyzed by two-way ANOVA (concentration x treatment).

**Fig 3:**
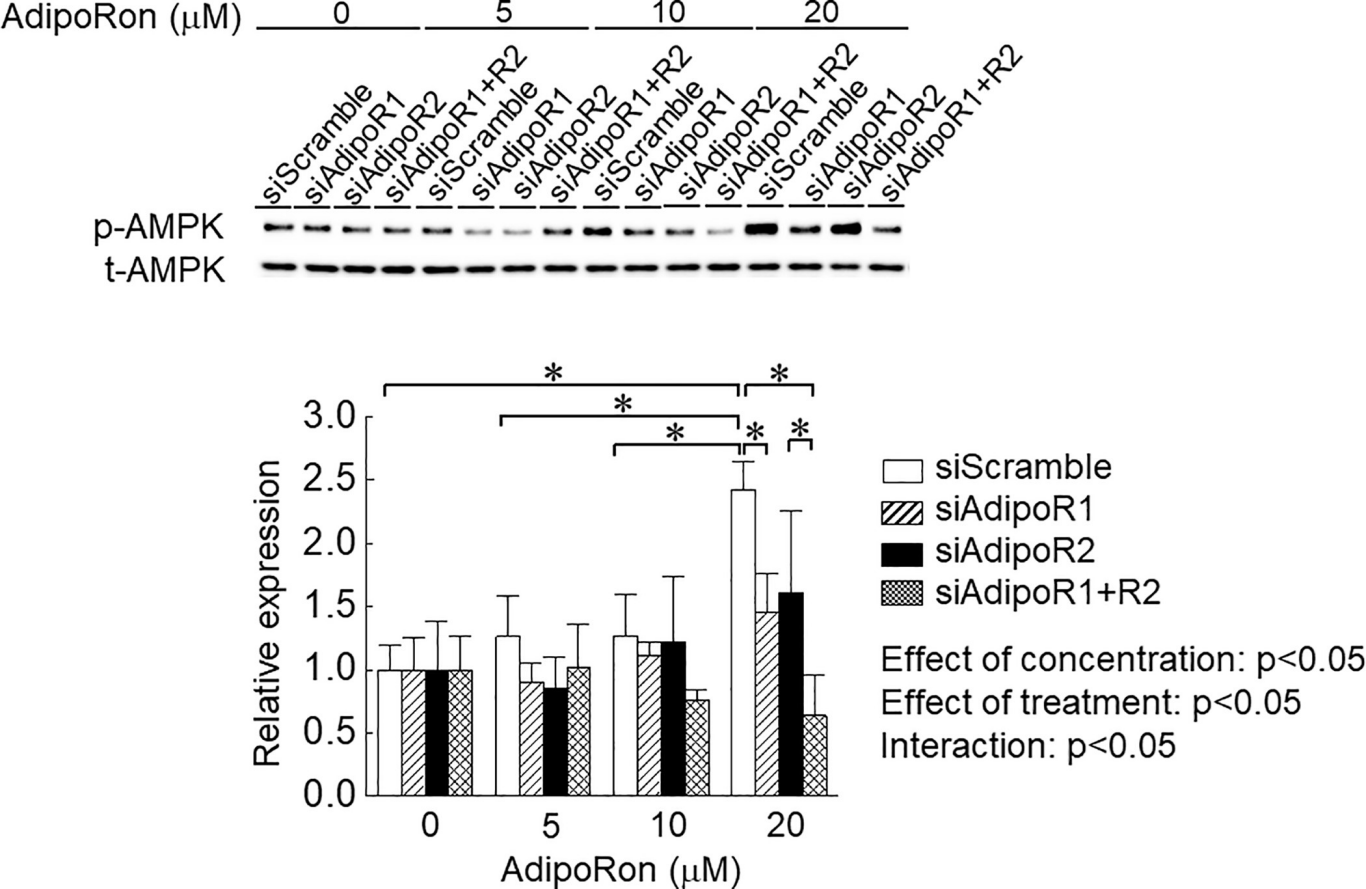
Effects of AdipoRon on phosphorylation level of AMP-dependent protein kinase (AMPK) with or without knockdown of adiponectin receptor (AdipoR). Abbreviations are the same as in Fig 1. n = 5 in each condition of each treated cells. Values are expressed means with SEM. *: p<0.05. Significant level of main effects was analyzed by two-way ANOVA (concentration x treatment).

**Fig 4:**
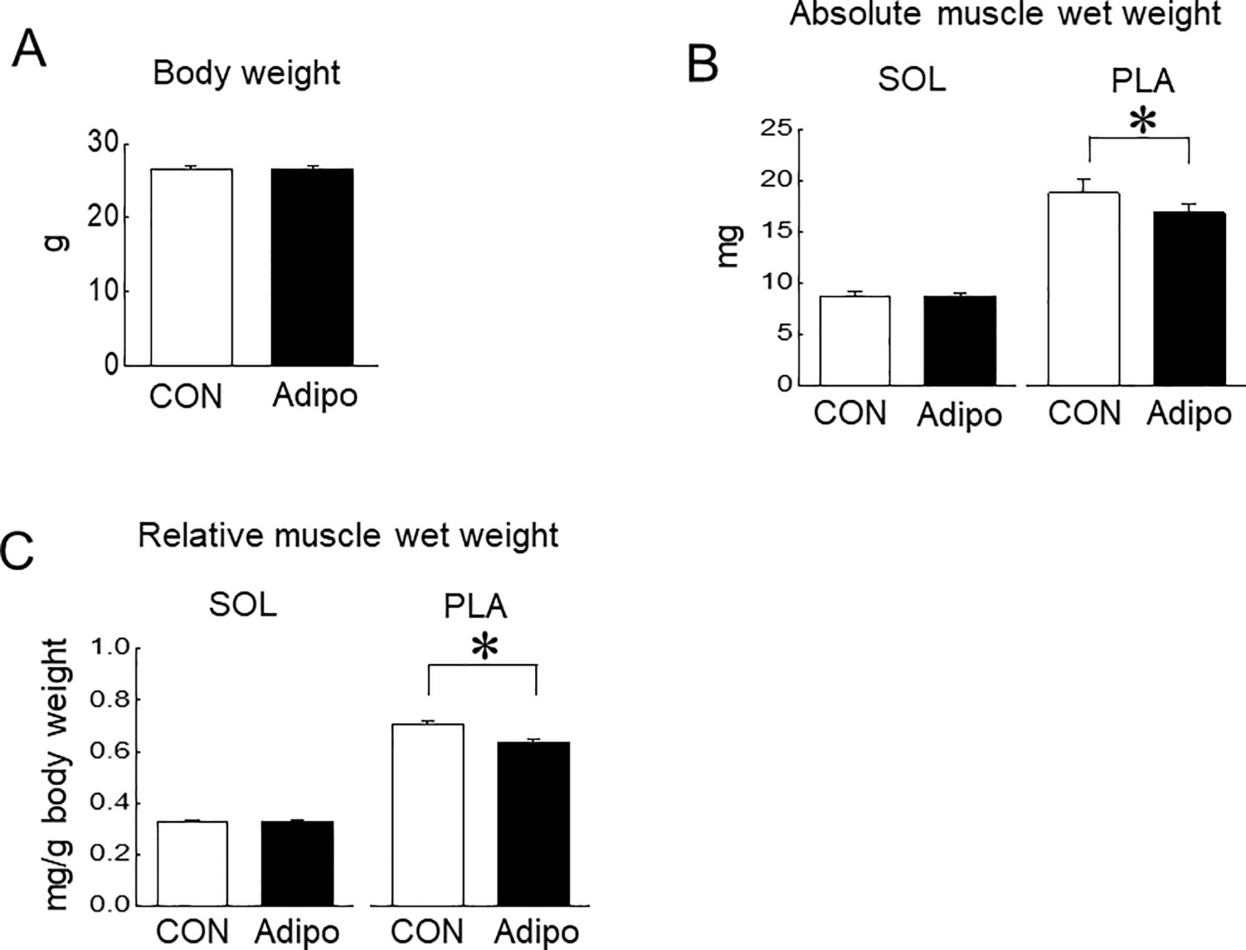
Effects of AdipoRon administration on mouse skeletal muscles. A: Body weight, B: absolute muscle wet weight of soleus (SOL) and plantaris (PLA) muscles, C: muscle wet weight relative to body weight. CON: vehicle-control group, Adipo: AdipoRon-administrated group, n = 7 and 5 in vehicle-control and AdipoRon-administrated groups, respectively. Values are means with SEM. *: p<0.05.

**Fig 5:**
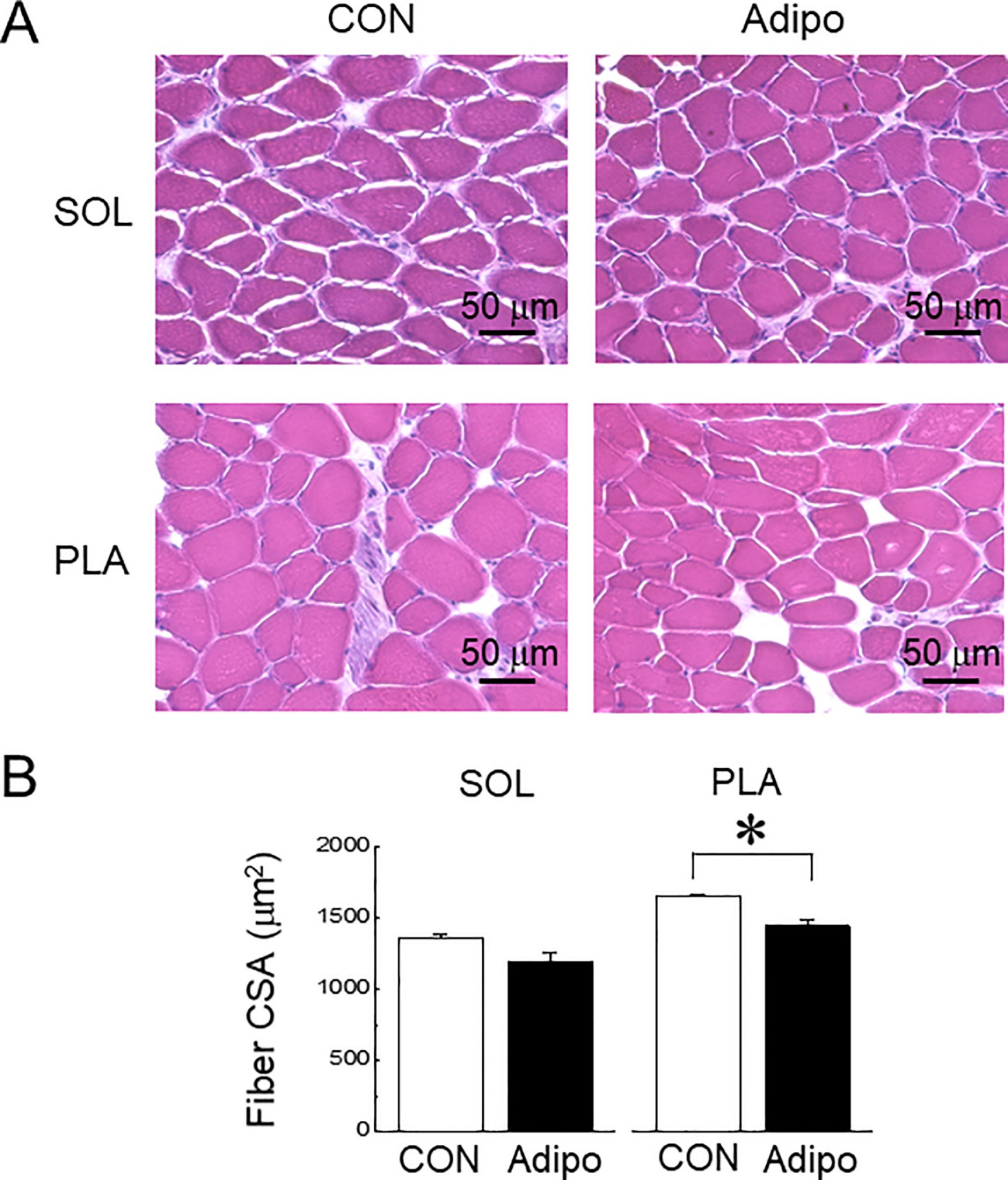
Effects of AdipoRon administration on fiber cross-sectional area (fiber CSA) of mouse skeletal muscles. A: representative images of H&E stained cross-sections of soleus (SOL) and plantaris (PLA) muscles, B: mean fiber CSA of SOL and PLA muscles. Abbreviations are the same as in Fig 4. n = 3. Values are means with SEM. *: p<0.05.

**Fig 6:**
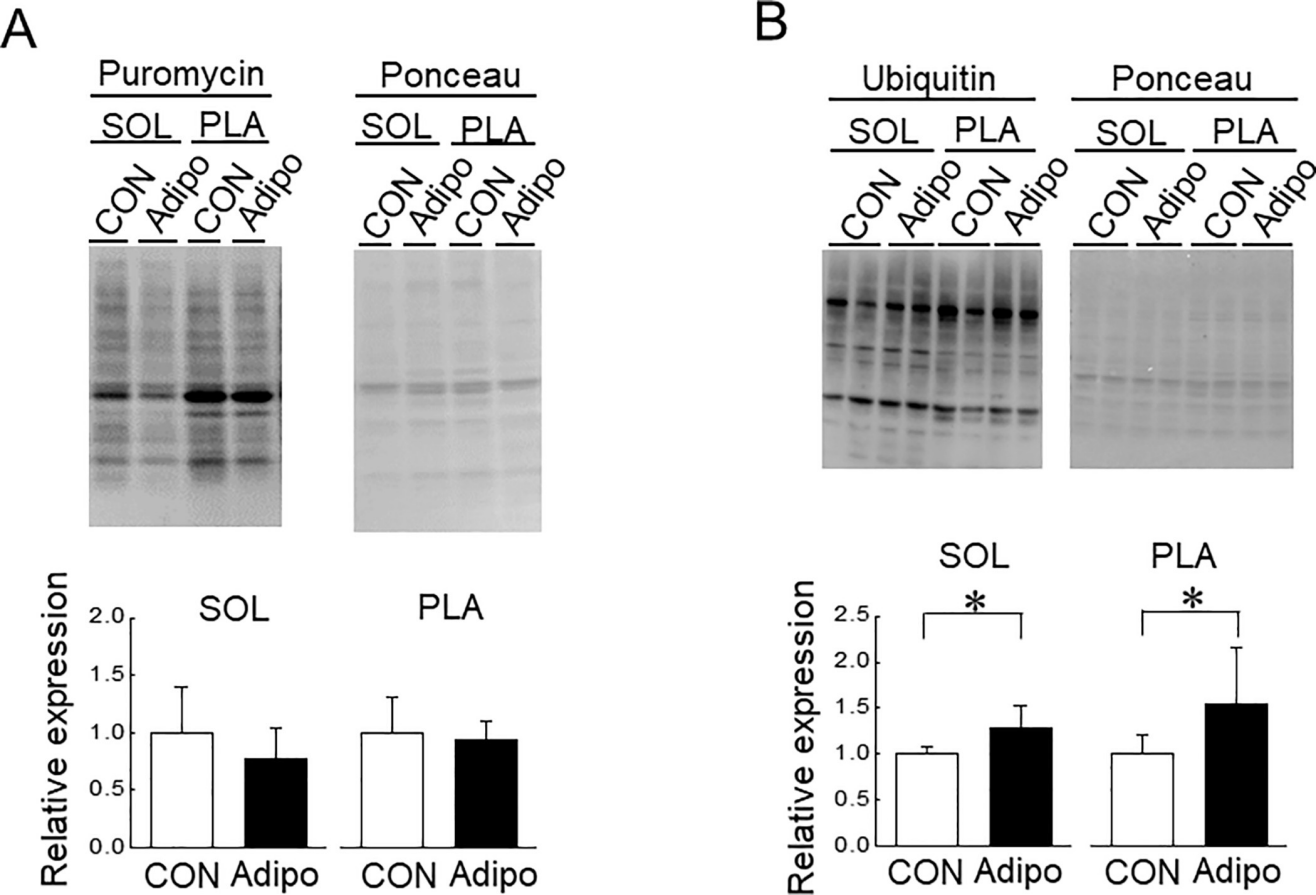
Effects of AdipoRon administration on the expression level of puromysin-labeled and ubiquitinated proteins in mouse skeletal muscles. A: Expression level of puromysin-labeled proteins, B: Expression level of ubiquitinated proteins. Ponceau staining was applied for the loading and blotting control of Western blotting analyses. Abbreviations are the same as in Fig 4. n = 7 and 5 in vehicle-control and AdipoRon-administrated groups, respectively. Values are means with SEM. *: p<0.05.

**Fig 7:**
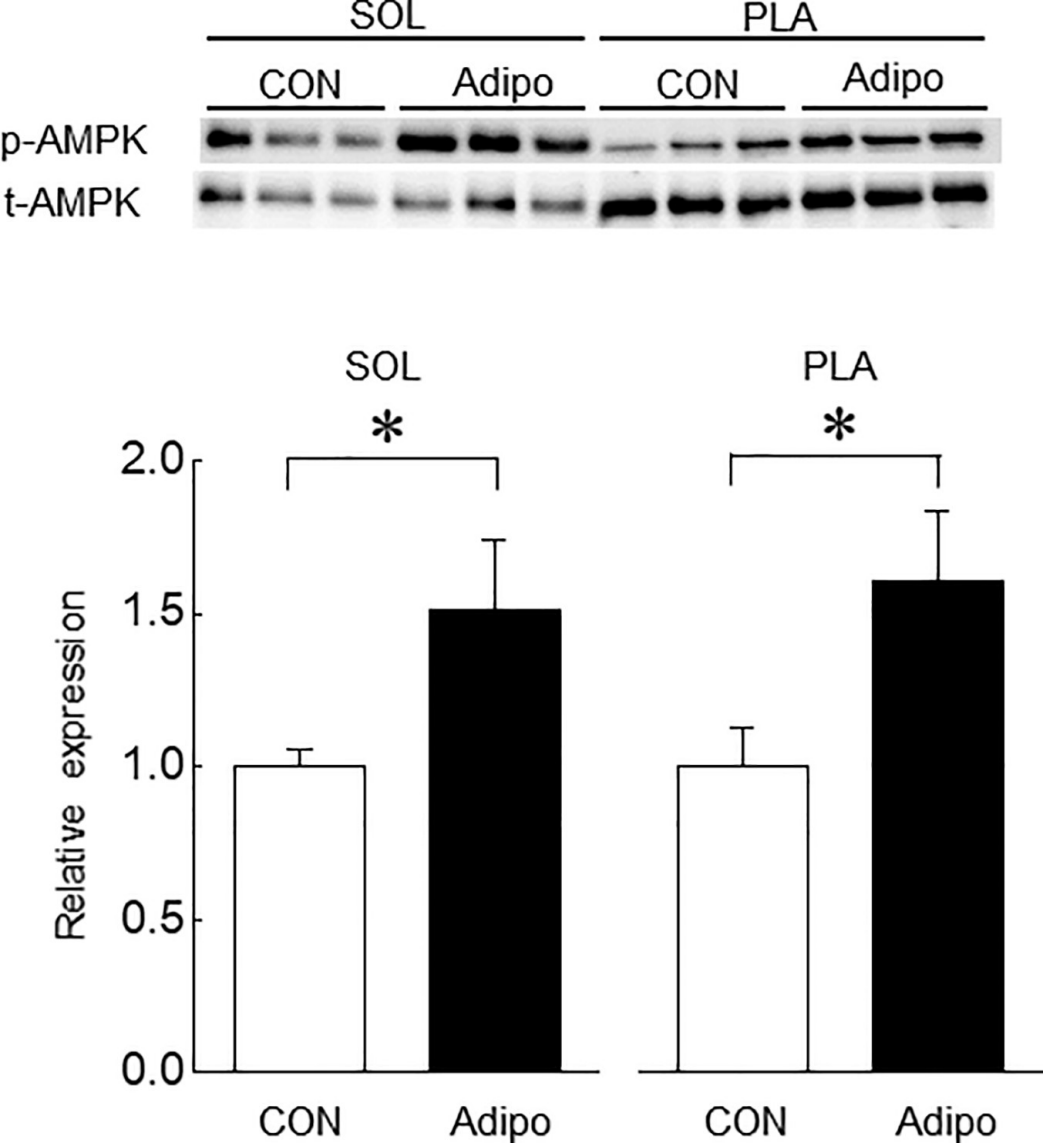
Effects of AdipoRon administration on phosphorylation level of AMP-dependent protein kinase (AMPK) in mouse skeletal muscles. Abbreviations are the same as in Fig 4. n = 7 and 5 in vehicle-control and AdipoRon-administrated groups, respectively. Values are means with SEM. *: p<0.05.

**Fig 8:**
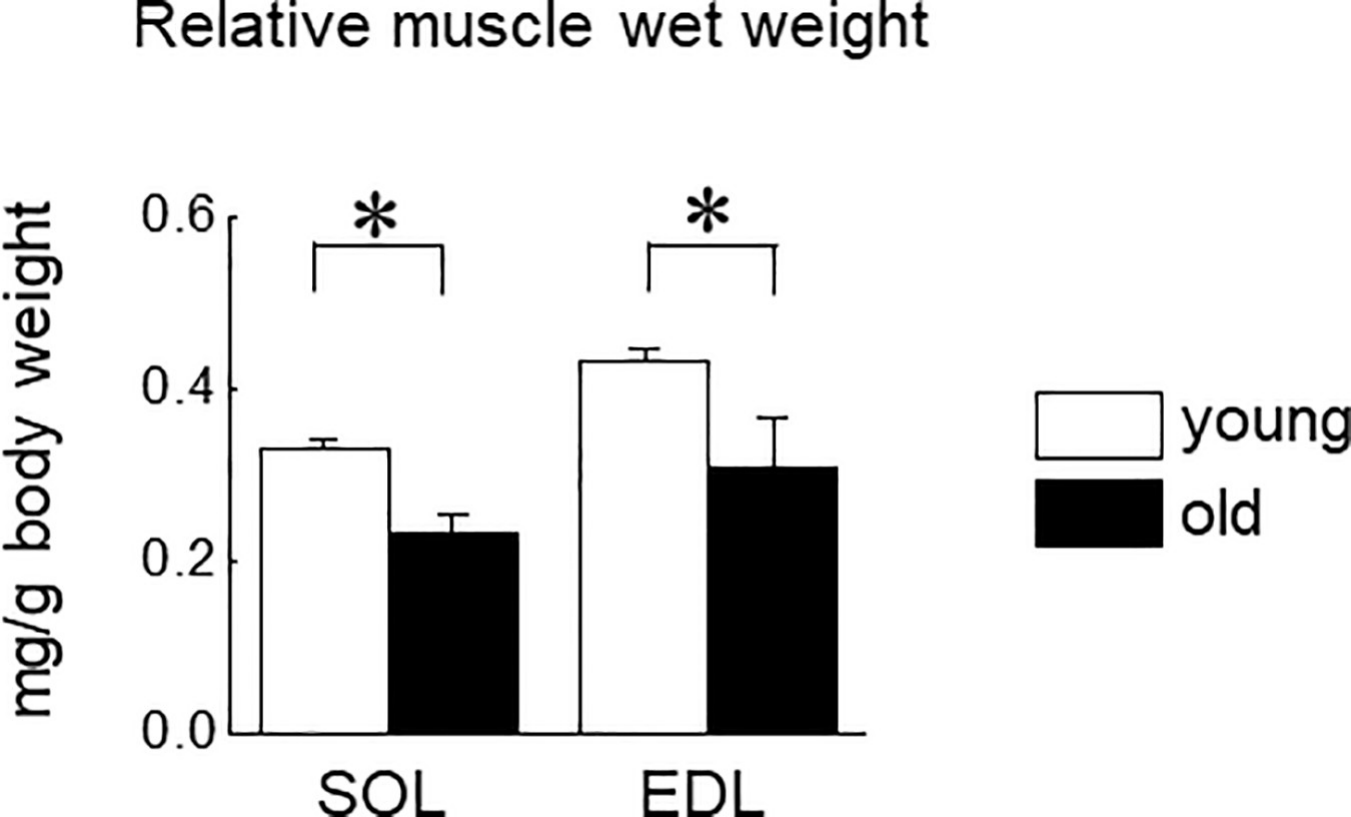
Muscle wet weight in soleus (SOL) and extensor digitorum longus (EDL) muscles of young and old mice. Young: 10-week old mice, old: 100-week old mice. n = 5 in each aged group of mice. Values are expressed means with SEM. *: p<0.05.

**Fig 9:**
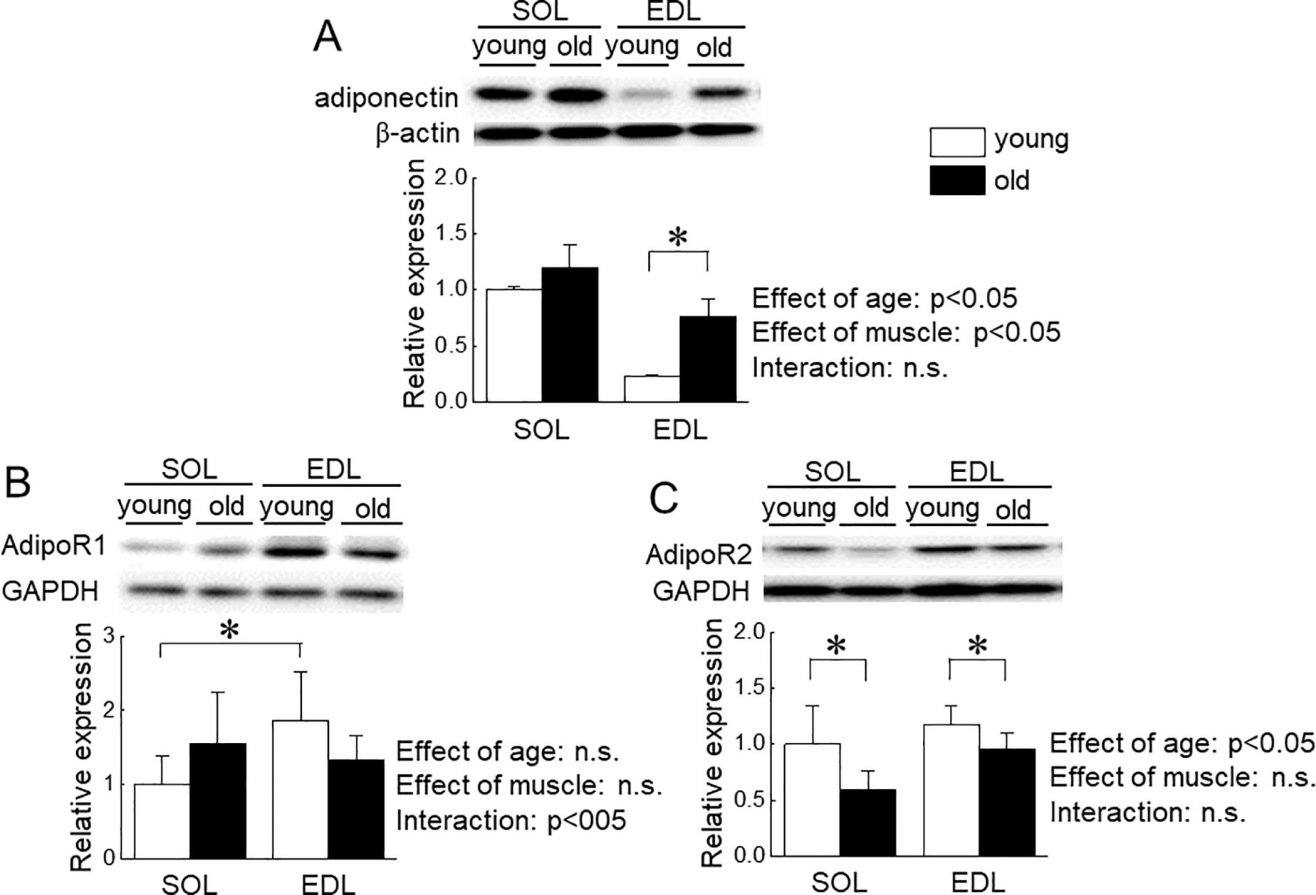
Expression level of adiponectin and adiponectin receptors in soleus (SOL) and extensor digitorum longus (EDL) muscles of young and old mice. A: adiponectin, B: adiponectin receptor 1 (AdipoR1), C: adiponectin receptor 2 (AdipoR2). Other abbreviations are the same as in Fig 5. n = 5 in each aged group of mice. Values are expressed means with SEM. *: p<0.05. Significant level of main effects was analyzed by two-way ANOVA (age x muscle).

**Fig 10:**
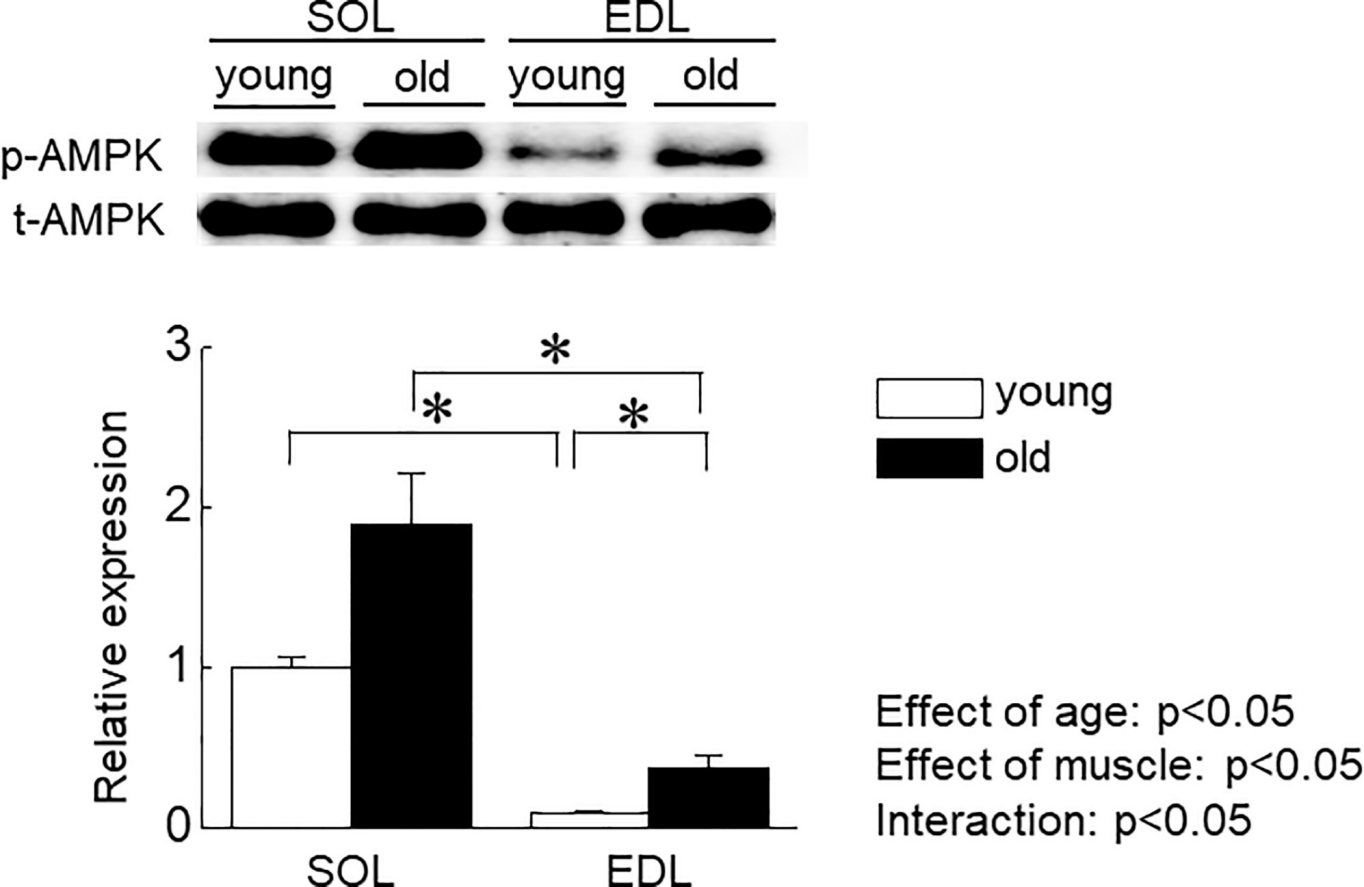
Phosphorylation level of AMP-dependent protein kinase (AMPK) in soleus (SOL) and extensor digitorum longus (EDL) muscles of young and old mice. A: adiponectin, B: adiponectin receptor 1 (AdipoR1), C: adiponectin receptor 2 (AdipoR2). Other abbreviations are the same as in Fig 9. n = 5 in each aged group of mice. Values are expressed means with SEM. *: p<0.05. Significant level of main effects was analyzed by two-way ANOVA (age x muscle).

All values were expressed as means ± SEM. Statistical significance was analyzed by using two-way ANOVA followed by post hoc comparison with Tukey’s test (Figs 1B, 2 and 3). Differences in the body weight, absolute and relative muscle wet weights (Fig 4), mean fiber CSA (Fig 5), the expression level of puromycin- and ubiquitin-labeled proteins (Fig 6), and the phosphorylation level of AMPK (Fig 7) were analyzed by using Student’s t-test. Deference in the muscle weights between young and aged mice (Fig 8) was also analyzed using Student’s t-test. Data in Figs 9 and 10 were analyzed by using two-way ANOVA followed by post hoc comparison with Tukey’s test. Data in S1 and S2 Figs were analyzed by one-way ANOVA followed by post hoc comparison with Tukey’s test. The differences between groups were considered statistically significant at p<0.05.

## Results

### Effects of AdipoRon on C2C12 myotubes

Fig 1 shows the effects of AdipoRon on C2C12 myotubes. Diameter and fusion index of myotubes was decreased by AdipoRon (Fig 2). However, knockdown of AdipoR1 and/or AdipoR2 partially rescued AdipoRon-associated changes of myotubes.

AdipoRon treatment decreased muscle protein content in a dose-dependent manner (Fig 1B; effect of concentration, p<0.05). Muscle protein content at 20 μM AdipoRon was significantly lower than that at 0, 5, and 10 μM AdipoRon (p<0.05, Fig 1B). AdipoRon-associated decrease in muscle protein content was partially rescued by knockdown of AdipoR1 and/or AdipoR2 (Fig 1B; effect of treatment, p<0.05). There was a significant difference in muscle protein content between siScramble and targeting siRNA for AdipoR (siAdipoR1 and/or AdipoR2, p<0.05).

Myotube diameter and number of nuclei per myotube was also suppressed by AdipoRon in as dose-dependent manner (Fig 2B and 2D; effect of concentration, p<0.05). In the present study, on the other hand, knockdown of AdipoR had no impact on both indexes (Fig 2A and 2C). AdipoRon-associated decrease in myotube dimeter and number of nuclei per myotube was partially rescued by knockdown of AdipoR1 and/or AdipoR2 (Fig 2B and 2D; effect of treatment, p<0.05). At 20 μM AdipoRon, there were significant differences in myotube diameter between siScramble and targeting siRNA for AdipoR (siAdipoR1 and/or AdipoR2, p<0.05).

AMPK is a downstream of adiponectin-AdipoR-associated signaling pathway [20,21], and is also known as a negative regulator for skeletal muscle mass [27]. Therefore, we investigated the effects of AdipoRon on phosphorylation level of AMPK in C2C12 myotubes. Phosphorylation level of AMPK showed a trend to be increased by AdipoRon (Fig 3). A significant increase in phosphorylation level of AMPK was observed at 20 μM AdipoRon, but not at 5 and 10 μM AdipoRon. Knockdown of AdipoR1 and/or AdipoR2 suppressed AdipoRon-associated upregulation of AMPK phosphorylation in C2C12 myotubes.

### Effects of AdipoRon administration on mouse skeletal muscles

Since the cell-culture experiments suggested that high concentration of AdipoRon causes to decrease in muscle mass, we investigated the effects of AdipoRon administration on mouse skeletal muscle mass *in vivo*. Successive intravenous injections of AdipoRon into mice caused a decrease in the absolute and relative muscle wet weights of PLA muscle (p<0.05), but not of SOL muscle, compared with the vehicle-control group (Fig 4B and 4C). However, there was no significant change in body weight (Fig 4A). Representative H&E images of SOL and PLA muscles were shown in Fig 5A. Mean fiber CSA in PLA muscle, but not in SOL muscle, was significantly decreased by AdipoRon administration (Fig 5B).

Then, we evaluated the levels of protein synthesis and proteolysis in SOL and PLA muscles using SUnSET method and ubiquitinated protein level, respectively (Fig 6A and 6B). A trend to suppress protein synthesis in AdipoRon-administrated PLA and SOL muscles was observed. However, AdipoRon administration had no significant effect on protein synthesis of both muscles. On the other hand, ubiquitinated protein expression in both muscles was significantly upregulated by AdipoRon administration (p<0.05, Fig 6B). We also investigated the effects of AdipoRon on phosphorylation level of AMPK in SOL and PLA muscles in mice. Phosphorylation level of AMPK in both muscles was significantly increased by AdipoRon administration (Fig 7).

### Expression level of adiponectin and its receptor in skeletal muscles in young and old mice

We investigated the effects of aging on the expression level of adiponectin and AdipoRs in mouse skeletal muscle. Both muscle weights were significantly decreased by aging (Fig 8, p<0.05). Basal expression level of adiponectin in SOL muscle was significantly higher than that in EDL muscle in young and old mice (p<0.05, Fig 9A). A significant aging-associated upregulation of adiponectin was observed in EDL muscle (p<0.05), but not in SOL muscle.

Fig 9B and 9C show the expression level of AdipoR1 and AdipoR2 in SOL and EDL in young and old mice, respectively. In young mice, the expression level of AdipoR1 in EDL muscle was significantly higher than that in SOL muscle (p<0.05). On the other hand, there was no significant difference in the expression level of AdipoR2 between SOL and EDL muscles. Aging-associated increase in the expression level of AdipoR1 was observed in SOL muscle (p<0.05), but not in EDL muscle. On the contrary, a significant decrease in the expression level of AdipoR2 was observed in both muscles (p<0.05). We also investigated the effects of aging on phosphorylation level of AMPK in SOL and EDL muscles in mice. In young mice, the phosphorylation level of AMPK in SOL muscle was significantly higher than that in EDL muscle (p<0.05). Phosphorylation level of AMPK in EDL muscles was significantly increased by aging, but not in SOL muscle (Fig 10).

## Discussion

This study investigated the effects of AdipoRon on the protein content of C2C12 myotubes and mouse muscle mass of slow SOL and fast PLA. AdipoRon suppressed the protein content, myotube diameter and number of nuclei per myotube of C2C12 cells in a dose-dependent manner via upregulation of phosphorylated AMPK. Adiponectin-associated decline of the protein content, myotube diameter and number of nuclei per myotube of C2C12 cells was partially rescued by knockdown of AdipoR1 and/or AdipoR2. Successive intravenous injections of AdipoRon into mice caused a decrease in the wet weight of PLA muscle, but not in SOL muscle. However, phosphorylation level of AMPK in PLA and SOL muscles was increased by AdipoRon administration. On the one hand, basal expression level of adiponectin in mouse slow SOL muscle was significantly higher than that in fast EDL muscle. On the other hand, the expression level of adiponectin in fast EDL muscle was lower than slow SOL muscle. Furthermore, the expression level of adiponectin and phosphorylated AMPK in EDL muscle was increased by aging, but not in SOL muscle. Aging had no impact on the expression level of AdipoR1 and AdipoR2 expression levels in both muscles.

This study demonstrated that AdipoRon administration caused a loss of muscle protein content, myotube diameter and number of nuclei per myotube of C2C12 cells as well as muscle wet weight and fiber CSA in mouse fast PLA muscle. This is the first study regarding the negative effects of AdipoRon on C2C12 myotubes as well as mouse fast-type skeletal muscle. Previous study showed globular adiponectin stimulated myogenic differentiation of C2C12 myoblasts under a starved condition that is serum-free growth medium [28]. Under starved condition, C2C12 myoblasts did not differentiate into myotubes [28]. In the present study, on the other hand, the effects of AdipoR agonist on C2C12 myotubes incubated with differentiation medium containing 2% horse serum. The differences in the culture conditions and/or in the timing of AdipoRon chemical may cause a conflict between 2 studies.

In the present study, knockdown of AdipoR1 and/or AdipoR2 partially rescued AdipoRon-associated decrease in the protein content, myotube diameter and number of nuclei per myotube of C2C12 cells. On the other hand, the phosphorylation level of AMPK was also depressed by knockdown of AdipoR1 and/or AdipoR2. It is also reported that globular adiponectin upregulated phosphorylated AMPK in C2C12 myoblasts [28]. Phosphorylated AMPK, which is active form, suppressed muscle protein content and myogenic differentiation of C2C12 cells [20]. In this study, AdipoRon administration also upregulated ubiquitinated protein level in mouse skeletal muscles. In addition, AdipoRon had no significant impact on protein synthesis in mouse skeletal muscles. Therefore, AdipoRon-associated decrease in muscle mass may induce by the enhanced proteolysis via activation of AdipoRs-AMPK signaling pathway.

Recently, a negative correlation between circulation level of adiponectin and muscle mass as well as and physical functioning in the elderly people, so-called adiponectin paradox, has been reported [12,13,14]. However, these reports are based on the epidemiologic cross-sectional studies. There were no experimental evidences regarding high level of adiponectin-associated skeletal muscle atrophy, namely adiponectin paradox in skeletal muscle. The present study demonstrated the first experimental evidences that the successive intravenous injection of AdipoRon into mice induces skeletal muscle atrophy. This phenomenon may be the first experimental evidence indicating “adiponectin paradox” in skeletal muscle. Since serum level of adiponectin is low in obesity, compared with non-obesity healthy people [2], it has been generally accepted that a high level of serum adiponectin gives us various benefits for our health, and has a preventive effect against life style-associated diseases [16,29]. However, a high level of serum adiponectin may impair skeletal muscle function.

In this study, AdipoRon-associated atrophy was observed in fast PLA muscle, but not in slow SOL muscle. There was no difference in AdipoRon-associated changes in proteolysis and protein synthesis of both type of muscles. Furthermore, phosphorylation level of AMPK in both muscles was also increased by AdipoRon. The molecular mechanism underlaying muscle type-dependent responses to AdipoRon remains unclear. We have no clear explanation regarding this discrepancy at present. Other proteolytic systems such as autophagy or calpain pathway might be involved in AdipoRon-associated fast type-specific skeletal muscle atrophy.

This study showed that the basal expression level of adiponectin in fast EDL muscle was lower than slow SOL muscle. Furthermore, aging-associated upregulation of adiponectin expression was observed in EDL muscle, but not in SOL muscle. On the other hand, there was no significant difference in aging-associated changes in AdipoRs expression level in both muscles. This is the first study showing muscle type-dependent difference in the expression level of adiponectin, and aging-associated upregulation of adiponectin in fast EDL muscle. It is suggested that inflammation upregulates adiponectin in skeletal muscle [16,29]. A possibility of inflammation in fast skeletal muscle, but not in slow muscle, might increase with aging.

Since skeletal muscle cells themselves synthesize and may secrete adiponectin in an autocrine- and/or paracrine-manner [15], the expression level of adiponectin in skeletal muscle might impact on skeletal muscle mass. In the present study, we observed aging-associated activation of AMPK in fast EDL muscle, but not slow SOL muscle. These results are consistent with the previous study [30]. Therefore, aging-associated upregulation of adiponectin in fast skeletal muscle may explain the molecular mechanism for sarcopenia, in part. Additional investigations are needed to elucidate this issue.

## Conclusion

AdipoRon caused a decrease in muscle protein content, myotube diameter and number of nuclei per myotube of C2C12 cells in a dose-dependent manner via AdipoRs-AMPK signaling pathway. Decrease in the wet weight of fast PLA muscle, but not in slow SOL muscle, was also induced by the successive administrations of AdipoRon into mice. Results from this study indicate that high level of circulating adiponectin may induce atrophy of fast type muscle. Aging-associated upregulation in skeletal muscle-specific adiponectin might be also involved in aging-associated skeletal muscle atrophy, namely sarcopenia.

## Supporting information

S1 Fig Knock-down efficiency of siRNA for adiponectin receptors (AdipoRs). A: Effects of single knockdown of AdipoR1 on AdipoR1 mRNA, B: Effects of single knockdown of AdipoR2 on AdipoR2 mRNA, C: Effects of double knockdown of AdipoR1 and AdipoR2 on AdipoR1 mRNA, D: Effects of double knockdown of AdipoR1 and AdipoR2 on AdipoR2 mRNA. Control: untreated control cells, mock-transfection: cells were treated with transfection reagents without siRNA, siScramble: scrambled non-targeting control siRNA, targeting siRNA: siRNA for AdipoR1 and/or AdipoR2. n = 5 in each condition of each treated cells. Values are expressed means with SEM. *: p<0.05.
(TIF)

S2 Fig Knock-down efficiency of positive control siRNA for glyceraldehyde 3-phosphate dehydrogenase (GAPDH). Abbreviations are the same as in S1 Fig. n = 5 in each condition of each treated cells. Values are expressed means with SEM. *: p<0.05.
(TIF)
