# Supplementary figures and images for: Activation of adiponectin receptors has negative impact on muscle mass in C2C12 myotubes and fast-type mouse skeletal muscle
Source: PLoS One. 2018 Oct 11;13(10):e0205645. doi: 10.1371/journal.pone.0205645 (PMC6181411; doi:10.1371/journal.pone.0205645)

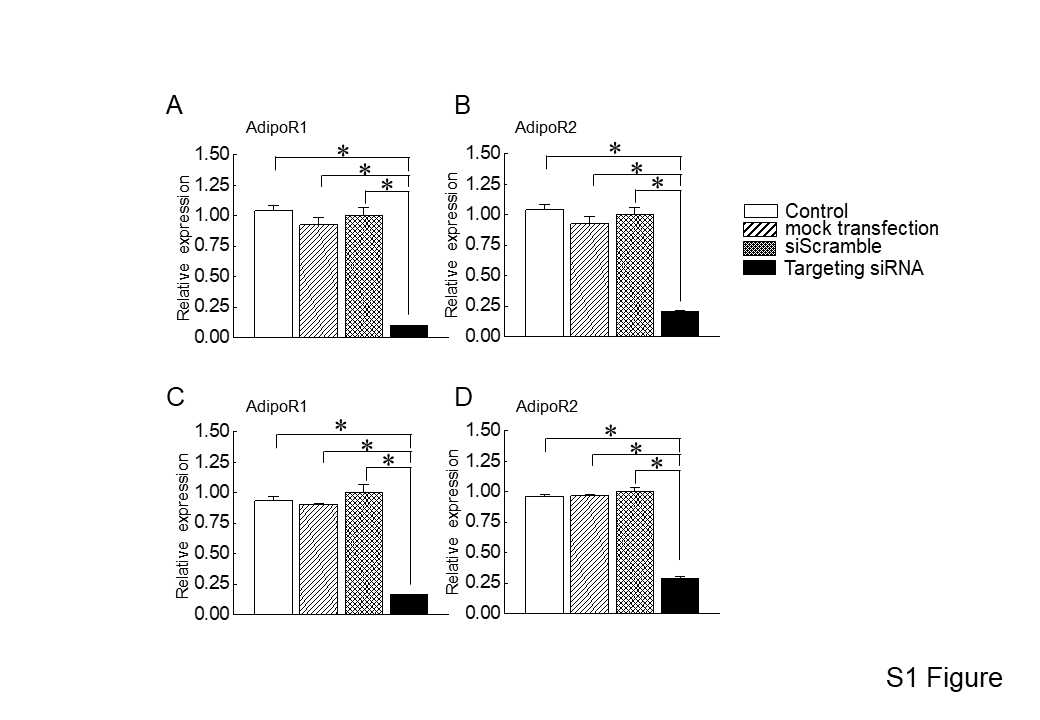

Supplement: S1 Fig — A: Effects of single knockdown of AdipoR1 on AdipoR1 mRNA, B: Effects of single knockdown of AdipoR2 on AdipoR2 mRNA, C: Effects of double knockdown of AdipoR1 and AdipoR2 on AdipoR1 mRNA, D: Effects of double knockdown of AdipoR1 and AdipoR2 on AdipoR2 mRNA. Control: untreated control cells, mock-transfection: cells were treated with transfection reagents without siRNA, siScramble: scrambled non-targeting control siRNA, targeting siRNA: siRNA for AdipoR1 and/or AdipoR2. n = 5 in each condition of each treated cells. Values are expressed means with SEM. *: p<0.05. (TIF) [file pone.0205645.s001.TIF]

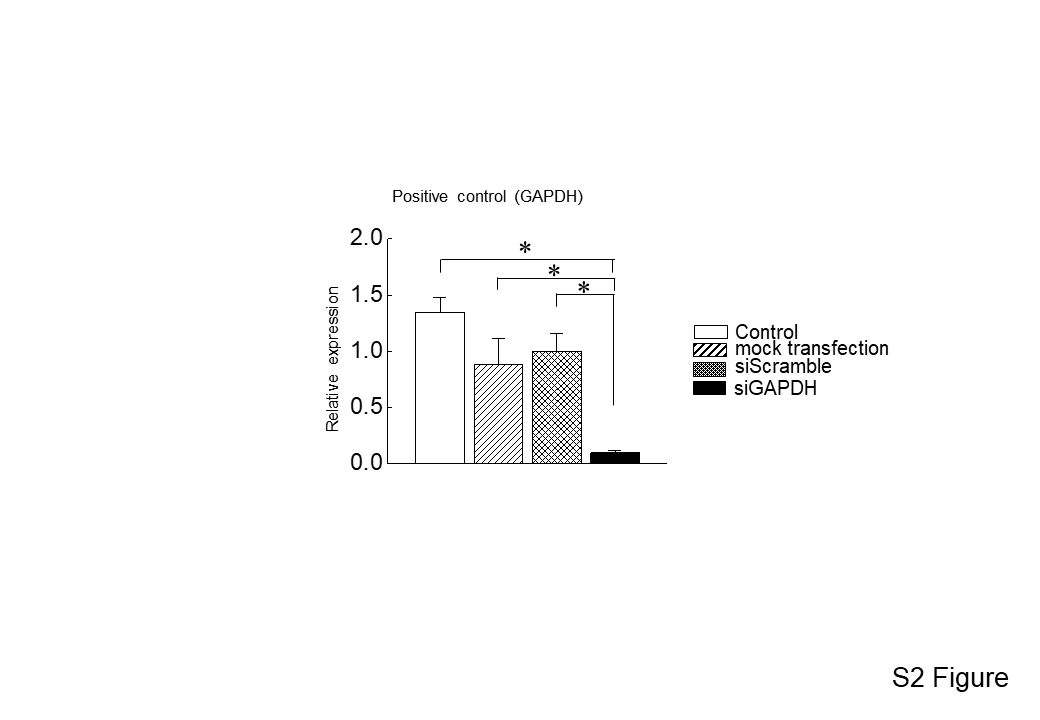

Supplement: S2 Fig — Abbreviations are the same as in S1 Fig. n = 5 in each condition of each treated cells. Values are expressed means with SEM. *: p<0.05. (TIF) [file pone.0205645.s002.TIF]
